# Chinese undergraduates’ English reading self-efficacy, intrinsic cognitive load, boredom, and performance: A moderated mediation model

**DOI:** 10.3389/fpsyg.2023.1093044

**Published:** 2023-02-03

**Authors:** Chenjuan Jiang

**Affiliations:** School of Foreign Studies, Jiangnan University, Wuxi, China

**Keywords:** reading self-efficacy, cognitive load, boredom, reading performance, mediation, moderation

## Abstract

**Introduction:**

Research findings from various academic domains have demonstrated that students’ self-efficacy (SE) influences their academic performance while limited studies have explored how foreign language reading SE influences reading performance. The purpose of this quantitative study was to explore the relationship between reading SE, intrinsic cognitive load (CL), boredom, and reading performance.

**Methods:**

The participants were 272 English-as-a-foreign-language (EFL) learners at a comprehensive university in China, who attended a compulsory English course for improving their English reading and writing proficiency. Data were analyzed through SPSS and structural equation modeling (SEM).

**Results:**

The result of the study provided support for the hypothesized relationships. Students’ English reading SE is positively related to their reading performance and negatively related to intrinsic CL. Their intrinsic CL during reading comprehension is negatively related to reading performance. Reading boredom is negatively related to reading SE and reading performance but positively related to CL. Furthermore, students’ CL mediates the relationship between reading SE and performance while the negative achievement emotion of boredom moderates the relationship between reading SE and CL.

**Discussion:**

The research highlights the importance of cognitive and emotional factors in influencing the relationship between foreign language reading self-efficacy and reading performance. Implications for EFL teachers and researchers are discussed.

## Introduction

Reading comprehension refers to “the process of simultaneously extracting and constructing meaning through interaction and involvement with written language” (RAND Reading Study Group, 2002). Success in school and future life greatly relies on the individual’s ability to read. Reading comprehension is a fundamental skill for university students to achieve success (Meniado, 2016). Foreign language reading is the basic content and form of foreign language learning as well as an important means for undergraduate students to broaden their knowledge. It has great value for them as a potentially rich source of input as well as enjoyment (Graham et al., 2020). In the reading process, many psychological and cognitive factors are found to influence reading performance; among these factors, self-efficacy (SE) and cognitive load (CL) as well as the emotional factor of boredom affect students’ performance of foreign language reading together. Therefore, foreign language reading is a complex and multidimensional process that has attracted the attention of many researchers.

## Literature review

### Reading self-efficacy and reading comprehension

Self-efficacy has been defined by Bandura (1986) as “People’s judgments of their capabilities to organize and execute courses of action required to attain designated types of performances.” This concept emerged from the social cognitive theory presented by Bandura (1986). SE for reading is defined as readers’ perceptions of competence in their capability to successfully complete reading tasks (Unrau et al., 2017). Students with relatively high SE are likely to manifest more reading achievement than students who doubt their ability to learn successfully (Bandura, 1997; Galla et al., 2014; Khamisi et al., 2016; Lee and Jonson-Reid, 2016; Forzani et al., 2021). Therefore, students’ SE beliefs are positively related to their academic performance (Bandura, 1986, 1997). It is often a better predictor of success than other factors such as prior accomplishments (Multon et al., 1991; Schunk, 1991). Most of the research found a positive and significant relationship between SE and reading performance (Khamisi et al., 2016; Shehzad et al., 2019a,b; An et al., 2020; Wang et al., 2021). Nevertheless, a few studies observed an insignificant association between these two variables (Wilson and Kim, 2016; Booth et al., 2017; Carroll and Fox, 2017). The contradictory results indicate that more research is necessary for English-as-a-foreign-language (EFL) countries where research regarding SE and reading comprehension is scarce (Shehzad et al., 2019b).

### Cognitive load and reading comprehension

The CL theory emerged as a major theory of cognition and instructional design (Ayres, 2006). Researchers have identified three kinds of CL: intrinsic, extraneous, and germane cognitive (Sweller, 1994; Sweller et al., 2019). Intrinsic CL represents “the inherent difficulty of the material itself” (Millin et al., 2020). It is the intrinsic characteristics of learning materials such as content that are difficult to learn (Chen and Lin, 2014). Extraneous CL refers to the load by the instructional design itself, and germane CL is the load placed on working memory during schema formation and automation. This research mainly focuses on intrinsic CL because we are only concerned about the contents and complexity of the reading materials, and reading comprehension is a kind of academic literacy skill, which tends to carry a heavy intrinsic load (Millin et al., 2020). EFL reading requires an individual to make an effort to understand the reading materials due to the intricacies of FL reading and the complex cognitive process (Graham et al., 2020). Chen and Lin (2014) found that the CL of learners in the low-cognitive-load group was significantly and positively correlated with their reading comprehension level. However, the study by Plassa et al. (2003) indicated that CL was negatively related to reading comprehension. Therefore, it is critical to conduct further research to explore the relationship between these two variables.

Instructional designers have frequently applied subjective measures as an instrument to estimate CL. Subjective measures are based on the assumption that learners are capable of reflecting on their cognitive processes and assessing the amount of mental effort used during finishing the task. Numerous pieces of evidence have supported this assumption, and subjective measures have been demonstrated to be highly reliable and more sensitive than physiological methods (Paas, 1992).

### Boredom and reading comprehension

The role of achievement emotion has been investigated widely in the school context. According to the control-value theory of achievement emotion, control, and value appraisals are the two determinants of achievement emotions (Putwain et al., 2018), among which boredom is a negative deactivating activity-related emotion (Pekrun and Perry, 2014) that has a detrimental effect on cognitive task performance because it decreases the availability of cognitive resources and fosters superficial information processing (Pekrun, 2006). Boredom can be conceptualized as an unpleasant emotional state, corresponding with low physical activation and cognitive situation, as well as specific perceptions and action tendencies such as escaping from ongoing boredom-eliciting situations through cognitive or/and behavioral disengagement (Goetz and Hall, 2014). Boredom experienced during a task increases distractibility and irrelevant thinking and leads to negative consequences in task performance (Zaccoletti et al., 2020). Some research has demonstrated a negative correlation between boredom and academic achievement (Zaccoletti et al., 2020). However, the scarcity of research into boredom in L2 teaching and learning has shown space for improvement and addition in future research (Li, 2021).

Until now, previous studies have shed light on how SE or CL, or boredom affect English learning performance. Nevertheless, no study has taken all the above variables into consideration in a structural equation model (SEM). To extend current research on what factors and how these factors affect students’ EFL reading performance, we used SEM to investigate the interplay of SE, intrinsic CL, and boredom that are related to reading comprehension performance. We, therefore, elaborated on the role that specific cognitive and emotional factors played in the EFL reading comprehension process.

## Research aim and hypotheses

Compared with the substantial studies investigating the relationship between SE and academic performance, there is still limited empirical evidence that addresses how foreign language reading SE influences foreign language reading performance. Most of the prior research has found a positive relationship between students’ SE beliefs and reading performance (Chapman and Turner, 1995; Wigfield and Guthrie, 1997). However, a few research indicated an insignificant relationship between these two variables (Eslami and Fatahi, 2008; Lau, 2009; Yilmaz, 2011; Wilson and Kim, 2016; Booth et al., 2017; Carroll and Fox, 2017). The inconsistent research findings make it necessary to conduct more research on the relationship between SE and reading performance. It was found that most of those studies were conducted in the United States. Shehzad et al. (2019a) pointed out in a systematic review that more research is necessary for those countries where research regarding SE and reading comprehension is scarce and more research needs to be conducted in EFL circumstances. The main purpose of this study was to investigate the relationships between English reading SE, CL, the negative achievement emotion of boredom, and reading performance in Chinese universities. Intrinsic CL was chosen as an important construct in the study because it has traditionally been known to affect students’ task performance (Yuan et al., 2006). We focus on the negative emotion of boredom because of the following two main reasons. (1) Boredom is a frequent emotion in the school context (Goetz and Hall, 2014). (2) The moderating effect of other negative academic emotions (anger, anxiety, shame, and hopelessness) has been confirmed to attenuate the positive influence of SE on academic performance in previous research (Villavicencio and Bernardo, 2013), while boredom has not been taken into account in the process of reading comprehension.

Previous research suggested that readers are more likely to make an effort and persist in reading a text if they believe in their ability to comprehend it successfully (Solheim, 2011), from which we can assume that reading SE can negatively predict the emotion of boredom. Empirical findings from previous studies conducted to date also showed that higher levels of boredom are associated with lower achievement (Daniels et al., 2009; Nakamura et al., 2021), which should include reading performance. Hence, based on relevant findings in previous studies, six hypotheses were proposed as follows:

H1: English reading SE is negatively related to CL.

H2: English reading SE is positively related to reading performance.

H3: CL is negatively related to reading performance.

H4: boredom is negatively related to reading SE and performance.

H5: CL mediates the effect of English reading SE on reading performance.

H6: boredom has a negative influence on the relationship between SE and reading performance by significantly moderating the mediating effect of CL.

## Materials and methods

### Participants

A convenient sampling method was used, and 274 non-English major sophomore students in a comprehensive university (a key university of Project 211) in a Chinese city participated in the project. However, the data of two respondents were eliminated from the database because their answers to all the survey questions are the same and they filled in the questionnaire in a very short time, which was impossible to finish completing the survey. The effective response rate was 99.3%. Among the 272 participants, 184 (67.6%) were men and 88 (32.4%) were women. The mean age of the participants was 19.64 (SD = 0.77) as shown in Table 1. All students were taking an English reading and writing course taught by the researchers of this study and gave their informed consent to participate in the study.

**TABLE 1:** Demographic information for a sample of second-year undergraduate students.

Variable	
**Age**	
Mean	19.64
Standard deviation	0.77
**Gender**	
Male	184 (67.6%)
Female	88 (32.4%)
Native language	Chinese
**Major**	
Science and engineering	225 (82.7%)
Liberal arts	47 (17.3%)

### Instruments

This research conducted one survey and one reading comprehension test for evaluating Chinese EFL learners’ reading SE, intrinsic CL, boredom, and English reading performance. The survey consists of three subscales and was measured with a seven-point Likert scale from 1 “strongly disagree” to 7 “strongly agree.” Since all the participants were EFL learners whose native language is Chinese, all the items in the survey were developed in Chinese. The following paragraphs give a brief description of the subscales of the questionnaire and the English reading comprehension test.

*Reading SE Scale* (Cronbach’s α = 0.781). After conducting a literature review on SE, a questionnaire was formed to assess the student’s English reading SE. Participants were asked to rate to what extent they were able to carry out a range of reading tasks by a seven-point Likert scale. Items were developed referring to Reading SE Scale in previous studies (Wang et al., 2014; KoşAr et al., 2022). After panel discussions by three expert teachers who specialize in teaching English reading and writing, three items were deleted, resulting in four items. An example item is “I can get a high mark in the reading comprehension part of the English test.”

*Reading Comprehension CL Scale* (Cronbach’s α = 0.881). The second questionnaire is to evaluate students’ intrinsic CL when completing the reading comprehension task. It is adapted according to the six dimensions of the NASA-Task Load Index (NASA-TLX) evaluation scale (Hart and Staveland, 1988), and four dimensions, namely, mental demand, time demand, effort, and frustration, were adopted. Four items are contained in the scale. An example item is “It took me a lot of effort to complete the reading comprehension test.” The scale showed acceptable reliability.

*Reading Boredom Scale* (Cronbach’s α = 0.829). Seven items measuring boredom were designed, and most of them were adapted from the boredom subscale of the Achievement Emotion Questionnaire (AEQ; Pekrun et al., 2005). The AEQ was originally developed in general educational psychology, allowing it to be applied in any specific domain. The adapted items were reworded concerning English reading in the present study. An example item is “When I am not interested in the genre of English articles, I feel that I am not willing to read it anymore.” The scale showed acceptable reliability.

*The Reading Comprehension Test*. A reading comprehension test was conducted to measure students’ English reading performance. The reading comprehension test was derived from the reading parts of four retired National College English Test Band 6 (CET-6) held from 2010 to 2012 to collect reading comprehension scores of the participant as CET is a nationwide proficiency test designed for non-English majors in tertiary education in China (Ying, 2020) and “the skills students reported in completing the CET reading are in line with the skills expected by the test designers” (Zheng and Cheng, 2008). Research indicates that the CET test is high in both reliability and validity (Lin, 2004). In the reading comprehension test, there were four passages followed by five multiple-choice questions, respectively, that asked students to make the right choice for each question. The score was five points for each question. The total score of the test was 100 points. The students were given 45 min to conduct the reading comprehension test and were informed that the reading comprehension test score would act as part of their normal performance score.

### Data collection procedure

Before data collection, researchers explained the purpose of the study to the participants and obtained their verbal consent. The data collection was conducted at the end of the academic year. First, the author uploaded the composite questionnaire to an online survey tool^1^ and retrieved its quick response code. Second, the reading comprehension paper test was administered among the participants during one of their English classes, the duration of which was 45 min. After the reading comprehension paper test, the assisting teachers presented the quick response (QR) code of the online survey to their students. The students then scanned the code with their mobile phones and got access to the online questionnaire. Approximately 7 min were given to complete the questionnaire.

### Statistical processing

SPSS 26.0 was used to conduct a common method bias test, independent-sample test, correlation analysis, and scale reliability analysis on the data. SEM with AMOS 24.0 was used to test the validity and reliability of the questionnaire items appearing in the SEM. To investigate the indirect effects of the SE variable through mediators, we followed the suggestions of Hayes (2009) and calculated the confidence interval of the lower and upper bounds to test whether the indirect effects were significant. A hierarchical moderator regression analysis was adopted to test the moderation effect of CL on the mediation model.

## Results

### Exploratory factor analyses of questionnaires

The questionnaire for evaluating students’ SE was self-designed, and the questionnaires for evaluating students’ CL and boredom are adapted according to the NASA-TLX evaluation scale and the boredom subscale of the AEQ, respectively. Therefore, we first performed the EFA to establish the factor structure of the questionnaire. We used the principal component analysis as the extraction method with the rotation method of varimax with Kaiser normalization (Kaiser, 1958). Following the principle stated by Stevens (1996), items weighted higher than 0.4 on the relevant factor were maintained.

Three factors with 14 items in total remained in the questionnaire (KMO = 0.868). The three factors were “SE” (Cronbach’s α = 0.781), “CL” (Cronbach’s α = 0.881), and “boredom” (Cronbach’s α = 0.794). The factor loadings of all items were higher than 0.6, from 0.62 to 0.82, suggesting the high validity of the measurement. The total variance explained was 61.175%. The overall Cronbach’s α is 0.633, presenting acceptable credibility of the measurement.

### Common method bias test

The Harman single-factor test was conducted on all the measured items in this study, and there were four factors whose eigenvalue was > 1. The first factor accounted for 37.22% of the total variation, <40% of the critical value, indicating that there was no serious common method bias in this study (Ylitalo, 2009).

### Correlation analysis

Means, standard deviations, and bivariate correlations for SE, CL, boredom, and reading performance are presented in Table 2. On average, participants reported moderate scores on all scales. It is obvious that all the variances are significantly related to each other. Reading SE was positively related to reading performance (*r* = 0.32, *p* < 0.01) and negatively related to CL (*r* = −0.36, *p* < 0.01). CL was negatively related to reading performance (*r* = −0.38, *p* < 0.01). Boredom is negatively related to both reading SE (*r* = −0.47, *p* < 0.01) and reading performance (*r* = −0.18, *p* < 0.01). Thus, Hypotheses 1–4 were confirmed.

**TABLE 2:** Correlation of main variables.

	Mean	SD	Min	Max	1	2	3	4
1. Self-efficacy^a^	4.06	1.11	1	7	1.00			
2. Cognitive load^a^	4.54	1.37	1	7	-0.36**	1.00		
3. Boredom^a^	4.40	1.10	1	7	-0.47**	0.44**	1.00	
4. Performance^b^	56.84	16.11	15	100	0.32**	-0.38**	0.18**	1.00
5. Skewness					-0.22	-0.18	-0.18	-0.09
6. Kurtosis					0.31	-0.28	0.64	-0.52

*n* = 272; SD, standard deviation; **p < 0.01 (2-tailed); ^a^possible range on self-efficacy (SE), cognitive load (CL), and boredom scales = 1–7; ^b^possible range of reading performance = 1–100.

The skewness and kurtosis and coefficient of SE, CL, boredom, and reading performance are also shown in Table 2. All the absolute values are <1, indicating that the data are approximate to normal distribution. Therefore, the maximum likelihood can be used.

### Confirmatory factor analysis of questionnaires

We then utilized confirmatory factor analysis (CFA) to further validate the instrument with questionnaires on SE, CL, and boredom. After the CFA, nine items remained in the finalized instrument. Three items remained on the scale of SE, three items remained on the scale of CL, and three items remained on the scale of boredom.

To measure the internal consistency reliability, convergent validity, and discriminant validity of the constructs in our proposed model, we performed CFA analysis on the constructs of SE, CL, boredom, and performance (see Table 3). The results revealed that the composite reliability of each construct ranged from 0.79 to 0.84, exceeding the 0.7 CR threshold value (Fornell and Larcker, 1981) and giving evidence of internal consistency reliability. In addition, the factor loadings of the individual items in the model were all significant (all *p* < 0.001). Meanwhile, the average variance extracted (AVE) of all constructs ranged from 0.56 to 0.64, exceeding the 0.5 AVE threshold value (Fornell and Larcker, 1981) and therefore the convergent validity was acceptable. Moreover, Table 4 shows that the estimated intercorrelations among all constructs were less than the square roots of the AVE in each construct, and this provides support for discriminant validity (Hair et al., 2006).

**TABLE 3:** Coefficient for the measurement model.

Construct	Items	Unstd.	SE	*t*-value	*P*	Std.	SMC	CR	AVE	Cronbach’s α
Self-efficacy	SE1	1.00				0.83	0.68	0.79	0.56	0.79
	SE2	0.77	0.08	9.17	***	0.64	0.41			
	SE4	0.83	0.09	9.79	***	0.77	0.59			
Cognitive load	CL1	1.00				0.83	0.68	0.84	0.64	0.84
	CL2	0.89	0.07	12.87	***	0.84	0.70			
	CL3	0.81	0.07	12.09	***	0.74	0.55			
Boredom	Boredom 1	1.00				0.89	0.79	0.79	0.56	0.78
	Boredom 3	0.81	0.09	9.23	***	0.72	0.51			
	Boredom 5	0.70	0.08	8.56	***	0.62	0.38			

*n* = 272; ***p < 0.001, SE, self-efficacy, CL, cognitive load.

**TABLE 4:** Discriminant validity of the constructs.

	AVE	Self-efficacy	Cognitive load	Boredom
Self-efficacy	0.56	(0.75)		
Cognitive load	0.64	−0.43	(0.80)	
Boredom	0.56	−0.59	0.32	(0.75)

Besides, following the principles of applying SEM in educational research, the structural modeling results (*x*^2^/df = 1.391, CFI = 0.993, IFI = 0.993, TLI = 0.988, SRMR = 0.030, RMSEA = 0.038, GFI = 0.983, AGFI = 0.959) also indicate that the hypothesized model fit the data well.

**FIGURE 1:**
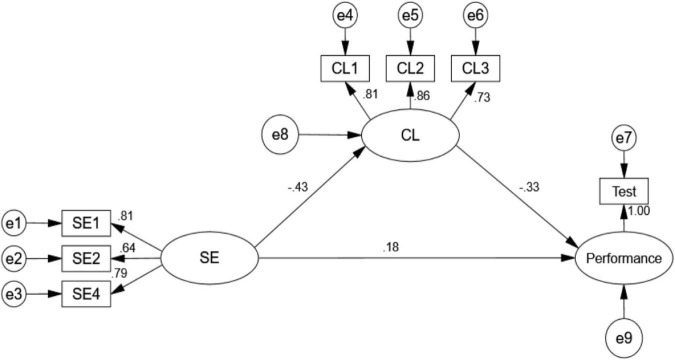
Structural equation modeling (SEM) of the hypothesized model. SE, self-efficacy; CL, cognitive load.

From Figure 1, significant negative effects of SE on CL, and CL on reading performance were observed. In addition, SE had a statistically significant positive effect on reading performance. Hypotheses 1–3 were thus supported again.

In order to prove the mediation effect of CL, we performed percentile bootstrapping and bias-corrected percentile bootstrapping at a 95% confidence interval with 10,000 bootstrap samples. We followed the suggestions of Preacher and Hayes (2008) and calculated the confidence interval of the lower and upper bounds to verify whether the indirect effects were significant. As shown in Table 5, the results of the bootstrap test confirmed the existence of a significant partial mediating effect of CL between reading SE and performance (indirect effect = 1.905, *Z* > 1.96, direct effect = 2.414, *Z* > 1.96, total effect = 4.32, *Z* > 1.96). Hypothesis 5 was thus supported.

**TABLE 5:** Unstandardized indirect, direct, and total effects of the hypothesized model.

	Point estimated	Product of coefficient		Bias-corrected		Percentile		Two-tailed significance
		SE	*Z*	Lower	Upper	Lower	Upper	
**Indirect effect**								
SE→Performance	1.91	0.56	3.43	1.05	3.34	0.94	3.11	0.00***
**Direct effect**								
SE→Performance	2.41	1.13	2.13	0.31	4.79	0.31	4.79	0.02*
**Total effect**								
SE→Performance	4.32	1.08	4.02	2.35	6.60	2.29	6.54	0.00***

SE, self-efficacy. ***p<0.001, *p<0.05.

We tested the hypothesized model with hierarchical moderator regression analysis by standardizing all variables to reduce the potential effects of multicollinearity and gender was used as a control variable because Pekrun (2018) suggested that learners’ emotions will demonstrate significant gender differences.

We tested the first-stage and second-stage moderation models as well as a direct-effect moderation model. The test involved the estimation of the following three equations:

First-stage moderation model:


(1) $$CL\;=\;a_{0}\;+\;a_{1}\text{SE}\;\text{+}\;a_{2}\text{BD}\;+\;a_{3}\text{SE}\;\times \;\text{BD}\;\text{+}\;e$$

Second-stage moderation model:


(2) $$\text{Performance}\;\text{=}\;b_{0}\;+\;b_{1}\text{SE}\;\text{+}b_{2}\text{CL}\;\text{+}\;b_{3}\text{BD}\;\text{+}b_{4}\text{CL}\;\times \;\text{BD}\;\text{+}\;e$$

Direct-effect moderation model:


(3) $$\text{Performance}\;\text{=}\;c_{0}\;+\;c_{1}\text{SE}\;\text{+}\;c_{2}\text{BD}\;\text{+}\;c_{3}\text{SE}\;\times \;\text{BD}\;\text{+}\;e$$

Table 6 shows that boredom moderated only the first stage path (*a*_3_ = 0.09, Δ*R*^2^ = 0.02, *p* < 0.05) but not the second stage path (*b*_4_ = 0.05, Δ*R*^2^ = 0, *p* > 0.05).

**TABLE 6:** Coefficient estimates for indirect effect in the first- and second-stage moderation model.

Variable	First stage (dependent variable = CL)				Second stage (dependent variable = performance)			
	Step 1		Step 2		Step 1		Step 2	
	*a*	*t*	*a*	*t*	*b*	*t*	*b*	*t*
Constant	-0.10	-0.57	-0.01	-0.08				
Gender	0.07	0.61	0.05	0.42				
SE	-0.23	-3.58***	-0.60	-3.81***				
BD	0.20	3.14**	0.19	2.94**				
SE × BD			0.09	2.56*				
Constant					-0.40	-2.39*	-0.42	-2.52*
Gender					0.30	2.54*	0.30	2.57*
SE					0.22	3.42**	0.21	3.17**
CL					-0.32	-5.45***	-0.34	-5.58***
BD					0.07	1.04	0.07	1.03
CL × BD							0.06	1.19
*R* ^2^	0.14		0.16		0.19		0.20	
*F*	14.73		12.91		15.82		12.96	
Δ*R*^2^			0.02				0.004	
Sig. Δ*F*			0.01				0.24	

SE, self-efficacy; BD, boredom; CL, cognitive load; ***p < 0.001, **p < 0.01, *p < 0.05.

In step 1 of the first-stage indirect effect moderation model, the CL was regressed to SE and boredom, and in step 2 the interaction item for SE and boredom was entered, *a*_3_ = 0.09, Δ*R*^2^ = 0.02, *p* > 0.05. The result shows that boredom did not moderate the indirect effect of SE on reading performance through the CL in the second stage.

In step 1 of the second-stage indirect effect moderation model, reading performance was regressed to SE, CL, and boredom, and in step 2 the interaction item for CL and boredom was entered, *b*_4_ = 0.06, Δ*R*^2^ = 0.2, *p* < 0.05. The result shows that boredom moderates the indirect effect of SE on reading performance through the CL in the first stage.

From Table 7, we can see that in step 1 of the direct effect moderation model, reading performance was regressed to SE and boredom, and in step 2 the interaction item for SE and boredom was entered, *c*_3_ = −0.1, Δ*R*^2^ = 0.03, *p* < 0.05. The result shows that boredom also moderates the direct effect of SE on reading performance.

**TABLE 7:** Coefficient estimates for direct effect in the moderation model.

Variable	Direct effect (dependent variable = performance)			
	Step 1		Step 2	
	*a*	*t*	*a*	*t*
Constant	-0.37	-2.10*	-0.47	-2.66**
Gender	0.28	2.22*	0.30	2.47*
SE	0.30	4.46***	0.74	4.61***
BD	0.00	0.02	0.02	0.28
SE × BD			-0.10	-3.03**
*R* ^2^	0.10		0.13	
*F*	10.11		10.10	
Δ*R*^2^			0.03	
Sig. Δ*F*			0.00	

SE, self-efficacy; BD, boredom; ***p < 0.001, **p < 0.01, *p < 0.05.

**FIGURE 2:**
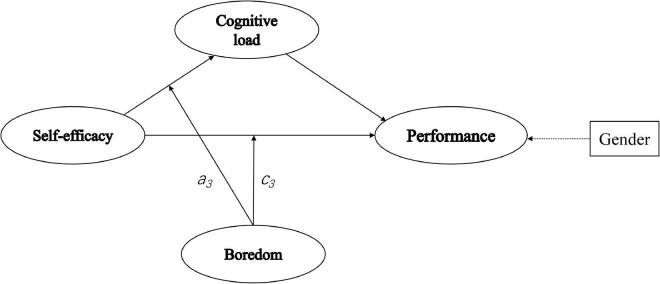
Moderated-mediation model.

In conclusion, our research proved that CL played a partially mediating effect between reading SE and reading performance. As can be seen in Figure 2, the mediating role of CL between reading SE and reading performance was moderated by the negative emotion of boredom, and the moderating role occurred in the first half of the path in the indirect effect and the direct effect.

The first-stage moderation effect was further analyzed by a simple slope test. For the moderating effect of boredom on reading SE and reading CL, boredoms were 0 (i.e., centered mean for boredom), −1.305 [i.e., one standard deviation (SD) below the mean], and 1.305 (i.e., one SD above the mean) for the mean, low, and high level, respectively. Simple slope (low boredom) = *a*_1_ + *a*_3_Boredom*_*low*_* = −0.718, simple slope (high boredom) = *a*_1_ + *a*_3_Boredom*_*high*_* = −0.494.

**FIGURE 3:**
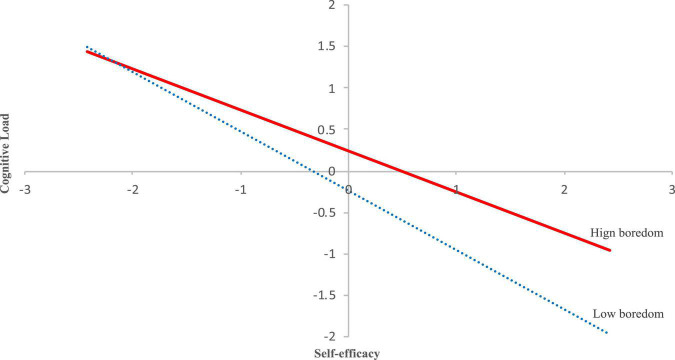
The moderating effect of boredom on self-efficacy (SE) and cognitive load (CL).

The result showed that compared with the reading SE of students with high reading boredom, the reading SE of students with low boredom had a more significant predictive effect on reading CL; that is, in terms of the effect of reading SE on reading CL, with the increase in the reading SE, both students with low and high reading boredom had a significant decrease in reading CL. Compared with students with high reading boredom, students with low reading boredom had a larger decrease (see Figure 3).

## Discussion

The current study explored the relationship between SE, intrinsic CL, boredom, and EFL reading performance of Chinese undergraduate students. The conduct of this research sheds new light on EFL learning in the following ways.

The first contribution of this research stems from the correlation analysis, which confirmed the findings of substantial studies that a positive and significant relationship between SE and reading performance (Bandura, 1997; Solheim, 2011; Su and Wang, 2012; Khamisi et al., 2016; Shehzad et al., 2019a,b; An et al., 2020; Soland and Sandilos, 2020; Forzani et al., 2021; Wang et al., 2021). This result is contrary to previous studies that have suggested an insignificant association between these two variables (Wilson and Kim, 2016; Booth et al., 2017; Carroll and Fox, 2017). Therefore, the findings of this study suggest that English teachers should not only pay attention to the training of students’ reading strategies but also attach significance to the training of their SE.

The second contribution of this research is the result that showed a negative association between intrinsic CL and reading performance, which is consistent with earlier research findings that students’ performance decreases with the increase in CL (Plassa et al., 2003; Yuan et al., 2006; Van de Weijer-Bergsma and Van der Ven Sanne, 2021).

The third contribution of this research is to examine the effect of reading SE on performance through the CL. SE had an indirect positive effect on reading performance through intrinsic CL and a direct positive effect on reading performance as well. This suggests that intrinsic CL partially mediates the positive relationship between reading SE and reading performance. The result is in line with the research finding of Van de Weijer-Bergsma and Van der Ven Sanne (2021) that CL mediated the relationship between personality and performance. According to this result, the ideal selection of reading materials is to optimize the intrinsic CL so that students can obtain the most information from the reading resources and work at full load in the reading process without affecting their SE.

The fourth contribution of this research is the incorporation of the moderating effect of the negative emotion of boredom on the relationship between SE and performance. This result confirms the research finding of Villavicencio and Bernardo (2013) that negative emotions moderate the relationship between SE and achievement. In this study, boredom moderates the SE and performance relationship through the mediation of CL, and the moderating effect only occurred in the first stage. With the increase in reading SE, students with low reading boredom had a larger decrease in CL. The result indicates that EFL instructors should identify and examine the factors that can be manipulated to help students reduce boredom feeling (Kruk et al., 2022) and to promote students’ positive emotional experience during the reading process.

The current study provides new insights into the relationships between reading SE and EFL reading comprehension performance by evaluating mediating and moderating effects involving two key factors, which are intrinsic CL and boredom. In addition to confirming the significant role of high levels of English reading SE in predicting increased levels of English reading performance, this study reduced some of the gaps in the literature by first testing the mediation of the SE and reading performance relationship by CL and the moderation of the SE and reading performance relationship by boredom, which have not been previously done. Through this research, we hope to have contributed a piece to the puzzle of the influence mechanism of reading in EFL.

## Conclusion

By exploring the relationship between English reading SE and reading performance, we have better chances of improving students’ reading comprehension proficiency by intervening in relative individual factors such as CL and negative emotion. The present study develops a moderated mediation model in which reading SE, intrinsic CL, and reading boredom are hypothesized to influence reading performance in a university context. Data were gathered from 272 students in different faculties in a comprehensive Chinese university. The analysis, through SPSS and AMOS, provided support for the hypothesized relationships. As indicated in this research, to improve students’ EFL reading performance, instructors can try to enhance students’ reading SE and meanwhile, optimize the CL of the reading materials, and reduce students’ feelings of boredom during the reading process.

## Limitations and future studies

We acknowledge some limitations of the present study. First, this study was quantitative in nature. Further research is encouraged to supplement the quantitative approach used in this study with qualitative procedures that may shed additional light on the process of understanding the antecedents of foreign language reading comprehension performance in higher education. Second, even though this study has shown that reading SE, intrinsic CL, and reading boredom are important factors explaining the difference in English reading performance, there are still many other factors that may affect foreign language reading performance, such as reading motivation, reading strategy, meta-cognitive awareness, English vocabulary, and other achievement emotions like enjoyment and anxiety. Future research could incorporate other variables in the influencing mechanism of reading comprehension.

## Data availability statement

The original contributions presented in this study are included in this article/supplementary material, further inquiries can be directed to the corresponding author.

## Ethics statement

The studies involving human participants were reviewed and approved by the Ethics Committee of Jiangnan University. Written informed consent for participation was not required for this study in accordance with the national legislation and the institutional requirements.

## Author contributions

CJ: data analyzing and interpreting, writing, reviewing, and editing.

## Footnotes

1
http://www.wjx.cn/
